# No Effect in Plasma Corticosterone Levels After Observation of Diseased Conspecifics

**DOI:** 10.1093/iob/obag008

**Published:** 2026-03-12

**Authors:** B M Hardy, M Chang, S McCallum, S Santangelo, P C Lopes

**Affiliations:** Schmid College of Science and Technology, Chapman University, Orange, CA 92866, USA; Schmid College of Science and Technology, Chapman University, Orange, CA 92866, USA; Schmid College of Science and Technology, Chapman University, Orange, CA 92866, USA; Schmid College of Science and Technology, Chapman University, Orange, CA 92866, USA; Schmid College of Science and Technology, Chapman University, Orange, CA 92866, USA

## Abstract

Organisms commonly respond to stressors in their environment by increasing the secretion of corticosterone (CORT). While increased CORT is acknowledged as a response to predation risk by organisms inhabiting a landscape of fear, new research raises the potential for changes in CORT as a response to the risk of infection by organisms in their environment. An increase in CORT of a healthy, uninfected individual in response to their perception of the risk of infection in their environment could change their susceptibility to infection, highlighting a significant role for CORT to alter host–pathogen dynamics. To investigate the effects of infection risk on host CORT levels, we conducted an experiment where healthy domestic canaries (*Serinus canaria*) observed canaries either infected with *Mycoplasma gallisepticum* (MG), which present behavioral and visual symptoms of infection, or symptom-free, sham-infected controls. We measured CORT from blood samples collected after either long-term (6 days) or short-term (1 h) observation of MG or sham-infected individuals. We found no difference in CORT levels between observers of MG or sham-infected birds at either time point, with the limitation of small sample size in the short-term observation (12 birds). We suggest further research should investigate changes in CORT at finer scales to ensure a complete picture of CORT profiles in response to infection risk.

## Introduction

Animals commonly respond to stressors in their environment by increasing the secretion of glucocorticoid hormones (e.g., cortisol or corticosterone; hereafter CORT) via activation of the hypothalamic-pituitary-adrenal (HPA) axis (Sapolsky et al. 2000). The activation of the HPA axis and short-term secretion of CORT mobilizes energy stores that may be beneficial for wound healing or escape behavior in response to predation, dangerous weather, or social conflict (Sapolsky et al. 2000). Animals not only increase their CORT in response to obvious stressors (e.g., predation or social conflict) but also respond to stressors that merely have the *potential* for negative fitness consequences. For example, many species exhibit higher CORT when inhabiting areas of increased predator density (Monclús et al. 2009), or simply seeing or smelling cues of predators without actual predation occurring (Cockrem and Silverin 2002; Muñoz-Abellán et al. 2009; Narayan et al. 2013). Exposure to predators or their cues can lead to sustained stress, with altered glucocorticoid secretion (Clinchy  et al. 2013). If predation risk [*sensu* (Laundré et al. 2001)] promotes stress responses in animals, then so too may the risk of infection from deadly pathogens or parasites (Weinstein et al. 2018).

Animals can perceive the risk of infection in their environment and can respond to this risk through a variety of ways (Lopes 2023), including behavioral avoidance (Buck et al. 2018) and physiological changes (Lopes 2022). There is even evidence for immune priming in anticipation of becoming infected (Love et al. 2021). It is possible that activation of the HPA axis with secretion of CORT during situations of high infection risk may be an important modulator of some of these physiological responses to infection risk. In one study, laboratory mice that had auditory, visual, and olfactory access, but not physical contact with blood-parasitized mice, displayed higher CORT than those with access to unparasitized controls (Curno et al. 2009). Researchers are also beginning to recognize the potential for “social stress transmission” in animal groups where a stressor-exposed individual generates a stress response in others via social information transfer from indirect or direct cues (Brandl et al. 2022). Under this scenario, the diseased individual would be the one under disease stress, and other animals would perceive the stress response and respond to it. This scenario could include amplification of stress across the animal group, acting as a “stress contagion” similar to expectations for pathogen transmission (Brandl et al. 2022). Regardless of the exact mechanism for a predicted increase in CORT in response to host perception of disease risk, there is potential for these predicted responses to affect disease outcomes.

Two key determinants of the effects of stress and CORT on animal immunology and disease outcomes are the duration and the intensity of the stress response (Dhabhar 2014, 2018). Prolonged/chronic stress or high intensity stressors are associated with immunosuppression/dysregulation and can increase disease susceptibility and severity, clinical signs of infection, and shedding of disease agents by, for example, decreasing leucocytes and increasing immunopathology (Biondi and Zannino 1997; Dhabhar and Mcewen 1997; Agarwal and Marshall 2001; Dhabhar 2009). Alternatively, CORT can actually increase immunoprotection during short-term/acute stress by increasing efficacy of healing and resisting infection, such as increasing heterophyl: lymphocyte ratios (Davis and Maney 2018), or reducing detrimental inflammatory immune responses (Sapolsky et al. 2000; Dhabhar 2009; Martin 2009). In fact, Adelman et al. (Adelman et al. 2015) found that pre-infection CORT was a significant predictor of eye pathology and sickness behaviors of house finches (*Haemorhous mexicanus*) infected with the bacterium *Mycoplasma gallisepticum* (MG). The authors found that higher circulating CORT was associated with lower eye pathology and sickness behavior once infected, and proposed that birds with higher CORT may be more tolerant to MG (Adelman et al. 2015). Another study using the same host–pathogen system found that, in males, higher pre-infection CORT concentrations were associated with reduced eye inflammation and pathogen load (Love et al. 2016). While no immune metrics were measured in these studies, it is well known that glucocorticoids can reduce inflammation in a context-dependent manner (Martin 2009). These results highlight the potential for CORT to play a significant role in host–pathogen dynamics and suggest that if CORT is increased as a response just from observing sick conspecifics, this could facilitate a positive feedback via increased tolerance in social songbirds.

The MG-songbird system is well-studied and one that is responsible for severe die-offs in wild populations since the 1990s when the pathogen emerged in house finches from poultry flocks (Faustino et al. 2004; Dhondt et al. 2005). Transmission is known to occur via direct bird–bird contact (Ley 2008), or indirectly via fomites on feeders, perches, or food (Dhondt et al. 2007). While MG can cause significant mortality in wild songbirds (Adelman et al. 2015), considerable intraspecific variation exists (Henschen et al. 2023), and recovery from infection can often occur both in the wild (Faustino et al. 2004) and in captivity (Kollias et al. 2004). Because MG causes obvious conjunctivitis (Kollias et al. 2004; Hawley et al. 2011), it is particularly suited to studying how hosts perceive infection risk. In fact, a recent study found that domestic canaries (*Serinus canaria*) observing MG-infected symptomatic conspecifics showed altered immune responses, including higher complement activity, when compared to canaries observing healthy controls (Love et al. 2021), an effect that could potentially be linked to altered CORT release. For example, it has been shown that immunoglobulins, complement protein levels, and neutrophil and monocyte populations can very rapidly (within minutes) increase after exposure to an acute stressor (Maes et al. 1997; Dhabhar 2018). Even though some mycoplasmas can escape the complement response, complement activity is important in the inactivation of mycoplasmas in general (Wang et al. 2023), and of *M. gallisepticum* in particular (Barker and Patt 1967).

We used a laboratory infection and observation experiment to test for differences in circulating CORT concentrations of domestic canaries (*S. canaria*) that observed MG-infected or healthy conspecifics. Because the duration of the CORT response is important to determine its immunological effects, we quantified CORT both after a short-term (1 h) and a long-term (6 days) exposure to infection risk. We predicted that both short-term and long-term observation of diseased conspecifics would lead to higher circulating CORT relative to observation of healthy individuals, similar to the effects of predation risk on CORT. Our study provides an essential first step to determine if host infection risk can alter circulating CORT, thus linking immune changes to the perception of infection risk.

## Material and methods

### Animals

Canaries were purchased as adults from Magnolia Bird Farms (Anaheim, CA, USA) and banded with individual numeric color leg bands upon arrival to our quarantine aviary space at Chapman University. Birds were housed for at least 1 month together in standardized conditions in the same room in groups of four to six in cages (You&Me™) prior to their use in the experiment. We provided water and food to birds (Leach Grain and Milling Canary Roller Mix^®^) *ad libitum*. Rooms were set on a 12:12 light dark cycle and received light from 7:00 to 19:00, and room temperature ranged between 18 and 21°C. Before the experiment, we took a blood sample (up to 70 μL) from the brachial wing vein into a heparinized capillary tube (Fisherbrand™ Microhematocrit Capillary Tubes, catalog # 22–362566). We used this blood sample to identify the sex of each individual genetically via polymerase chain reaction (PCR) and test for evidence of MG infection via detection of antibodies (see *Laboratory Assays*). Birds were not molting or in breeding condition during the experiment (see details below).

### Experimental design

The experiment consisted of two treatments: observers of MG-infected or sham-infected (details below) stimulus birds either for 6 days (Chronic) or 1 h (Acute; consisting of one overnight in darkness and one daylight hour) (Table 1). We note that this is an opportunistic study that takes advantage of the availability of plasma from two separate larger experiments (hence, the difference in sample size between the Chronic and Acute treatments). The sampling days for the Chronic and Acute treatments were selected to coincide with the days where eye pathology of infected birds was likely at its peak (D6 and D7 post-infection, respectively). Furthermore, the Chronic time sample of D6 falls within the time points where the Love et al study (2022) found differences in immune responses between observers of MG vs. SHAM treated birds. The Acute timeframe of 1 h of visualization of the stimulus birds reflects the fact that CORT increases very rapidly in response to stressors, peaking between 30 and 60 min and returning back to baseline at 2–3 h after onset, though this timeframe is variable and depends on various factors, including stressor type (Herman et al. 2016).

**Table 1:** The sample sizes of female (F) and male (M) canaries that observed each stimulus (sham or MG exposure) for each treatment (Chronic or Acute)

Treatment	Observed MG-stimulus	Observed sham-stimulus
Chronic	10F, 10M	10F, 10M
Acute	6F, 6M	6F, 6M

Two adjacent experimental rooms were used for the experiments. Each room contained two observer shelving units with two levels of racks that could accommodate between six and eight cages. Directly across from the observer racks sat corresponding stimulus cages 2.4 m away. This is the same distance used by the Love et al. (2021) study, confirming observers are close enough to observe focal individual symptoms. The sides of observer cages were blocked in a way that prevented each observer from seeing any neighboring observers thereby limiting their view to only stimulus birds. Observer birds were housed individually and, therefore, the only birds that observers had visual access to were the stimulus birds. In each room, six or eight observers were allowed visual and auditory access to three or four female stimulus birds, respectively [following (Love et al. 2021)]. Only a single stimulus treatment (MG or SHAM infected) was present in an experimental room at a given time.

Animals were monitored daily throughout the experiment to refill food and water containers and assess general animal health. Experimental rooms were set under the environmental conditions (same dark light cycle and temperature) as the quarantine room. To reduce the possibility that observers would be exposed to MG, two measures were taken: a clear Plexiglass barrier was placed on the side of the MG treated birds that faced the observer birds, and an air purifier with high-efficiency particulate air (HEPA) filtration (Levoit^®^ Vital 200S-P) was present in each experimental room. A similar Plexiglass barrier was successfully used by other researchers and still elicited responses in observers (A. Love personal communication). This experiment was approved by Chapman University Institutional Animal Care and Use Committee (approval no. 2022–1198) and was conducted according to the Association for the Assessment and Accreditation of Laboratory Animal Care Guidelines.

### Chronic observation experiment

Birds for the chronic experiment were moved from the aviary into new cages (Seny^®^) in the experimental rooms 3–5 days prior to the start of the experiment. At the start of the experiment on D0, we scored each eye on a scale of 0–3, in 0.5 increments, to describe the severity of any eye inflammation (eye pathology) following a previously published protocol (Love et al. 2021). Scores from each eye were summed to provide a single value per individual on a scale of 0–6. Stimulus birds were then exposed to either an MG solution in Frey’s media (2.80 × 10^6^ CCU/mL concentration; strain VA1994 (stock ID 7994–1(6P), Steven J. Geary University of Connecticut; MG treatment) or MG-free Frey’s media (sham treatment). Exposure consisted of 20 μL of solution pipetted into each eye. Observer birds observed the stimulus birds until day 6 post stimulus treatment (D6). To determine if chronic visual exposure of observing diseased stimuli also affected uninfected observer weight, we weighed observers on D0 and D5 to record any changes in mass. Weighing was done in the morning, on an Escali L600 High Precision Digital Scale. To document disease progression and effects on stimulus birds, we scored eyes again on D3, and D6 and weighed stimuli on D0 and D6. On D6, observers were quickly euthanized via isoflurane inhalation and decapitation outside of the experimental room. Blood from the trunk was then collected into Ethylenediaminetetraacetic acid (EDTA)-treated tubes and placed on ice within 12 min of initially entering the room and <3 min from capture, handling, and euthanasia of individual birds. Blood samples were then centrifuged within 1 h of collection to separate blood from plasma, and samples were stored at −80°C. Refrigerated steroids are known to be stable for days (Olson et al. 1981; Reimers et al. 1983; Bennett et al. 2012; Khonmee et al. 2019).

To document that chronic observers did not become infected with MG during the experiment, we scored eyes of observers on D5, and we took eye swabs from observers immediately post-mortem to test for cross-contamination of MG during the experiment. Both eyes of each observer were swabbed with sterile cotton swabs dipped in tryptose phosphate broth (TPB, Gibco^TM^, catalog #18050039) to test for inadvertent MG infection during the experiment. We used one swab per eye and rotated the swab across the conjunctiva for 5 s before placing the swab into the TPB-filled tube. The swab used for the second eye was also placed in the same tube.

### Acute observation experiment

Following the completion of the chronic experiment, stimulus birds were retained in the experimental rooms, and new observers for the acute experiment were brought into those rooms at 19:00 on D6 when the lights had already turned off. The acute observers were transported at this time to the experimental room to allow for acclimation and reduce any transport stress response prior to observing the MG-infected stimuli. Therefore, the acute timeframe for observers reflects one overnight in darkness (obstructing clear visual observation) and 1 h of light with the MG-infected animals. Experimental room darkness prevented the observers from visualizing symptomatic MG-infected or sham-treated stimuli until 7:00–8:00 on D7 when the lights turned on. After 1 hr of observation, acute observers were then euthanized, blood collected within 6 min of initially entering the room, and eyes swabbed identically to the chronic experiment as described above.

### Laboratory assays

#### Genetic sexing

We extracted DNA from whole blood samples with Zymo™ Quick-DNA Miniprep Plus^®^ Kit (#D4069). Extracted DNA was quantified on a Thermo Scientific™ Nanodrop 2000^®^ prior to PCR. For the PCR reaction mix, we used the P2 and P8 primers described in (Griffiths et al. 1998), in 50 μL reactions containing 200 ng of DNA, 2 μL of each 10 μM primer, 5 μL of 2 nM dNTPs, 0.25 μL of DreamTaq DNA polymerase (Thermo Scientific™, catalogue #EP0703), 5 μL of DreamTaq buffer, and DNase-free water. The cycling conditions were 95°C for 3 min, followed by 40 cycles of 95°C for 30 s, 48°C for 45 s, 72°C for 45 s, with a final extension step of 72°C for 5 min. PCR products were run on a 2% or 4% agarose gel to determine sex banding pattern. Sex was also confirmed by gonadal inspection postmortem, showing 100% match with the genetic sexing, and that gonads were small or under-developed.

#### MG antibody

We tested for the presence of MG-antibodies in canaries prior to the start of our study using the IDEXX™ MG Ab Test Kit (#99–06729), following manufacturer’s instructions with minor modifications. Namely, a blocking step of 40 min at room temperature was added at the start of the protocol, by applying 300 μL of 1% normal goat serum in phosphate-buffered saline to each well and samples were diluted 1:50 in sample diluent prior to being assayed. Plasma samples were run in singlicate on two plates and read on a Tecan Spark^®^ Multimode Microplate Reader at 650 nm. Sample values were calculated by first subtracting the mean negative control absorbance from the mean positive control absorbance and from each sample absorbance, and then by dividing the corrected sample absorbance by the corrected mean positive control absorbance. Samples were considered positive for MG antibodies if the calculated sample value was larger than 0.5, and negative if equal or smaller than 0.5, as per manufacturer’s guidelines.

#### CORT

We quantified CORT from diluted plasma samples (1:30 dilution) using Arbor Assays™ DetectX^®^ Corticosterone Enzyme Immunoassay Kit (#K014-H1/H5). We followed the manufacturer’s protocol for the 50 μL assay format with one modification: the standard curve followed the concentrations used for the 100 μL assay format as they better fit the range of the CORT concentrations of our samples based on preliminary testing. Our preliminary testing of this kit, under identical conditions to the two experimental sample plates, confirmed that MG-infected stimulus birds indeed displayed increased CORT (40.2 ng/mL) compared to controls (6.02 ng/mL), showing that changes in CORT are detectable with this assay. All samples and standards were run in duplicate. Plates were read on a Tecan Spark^®^ Multimode Microplate Reader at 450 nm. After subtracting sample and standard absorbances from the mean non-specific binding well absorbances, CORT concentrations were interpolated from the standard curve using a 4-parameter linear regression. Samples from the Chronic treatment were all on a single plate and those from the Acute treatment were distributed across two plates. Average intra-plate variation was 0.03% and average inter-plate variation was 0.04%.

#### Real-time quantitative PCR detection of MG in eye swab samples

We extracted DNA from eye swabs using the Zymo™ Zymobiomics Microprep^®^ Kit (#D4301) according to the protocol for swab samples with the following alterations: we heated the elution buffer to 60°C prior to elution and let elution buffer sit on the filter media for 5 min instead of 1 min. We then prepared qPCR reactions to test for the presence or absence of MG in our sample, using the same *mgc2* primers, and similar reaction mix and cycling conditions as in (Hawley et al. 2011). Each reaction consisted of 10 μL of SSO Advanced Universal SYBR (Bio-Rad, catalog #1725275), 1 μL of each primer (at 10 μM concentration), 3 μL of sample DNA, and 5 μL of DNase-free water, for a total volume of 20 μL. Quantitative real-time was run on a CFX instrument (Bio-Rad), and cycling parameters were 98°C for 3 min, followed by 40 cycles of 98°C for 10 s and 60°C for 30 s. A cycle threshold (Ct) value of less than 40 was the cutoff for samples to be considered positive for *M. gallisepticum* [as in (Hawley et al. 2011)]. The only sample to meet this cutoff was a sample from an experimentally infected animal (Ct = 17.93), used as a positive control.

### Data analysis

We first tested for any correlations by implementing a correlation test using Pearson’s correlation coefficient between CORT and the time elapsed since initial room entry and the blood sample taken (1–12 mins for Chronic and 1–6 mins for Acute) and found no influence of sampling time on CORT measures (Figs. S1–S2). Given that CORT did not change with time, we used all samples collected. We also tested for any changes in individual body mass in the chronic experiment prior to our formal analysis to ensure it did not need to be accounted for as a nuisance variable, and found no changes in body mass using an aligned ranks transformation analysis of variance (ANOVA) for non-parametric data (Fig. S3). Our measure of CORT was square-root transformed to meet requirements of normality and homoscedascity for the chronic experiment dataset. The residuals of the acute experiment dataset were normally distributed and met the assumption of homogenous variances. We used linear models (two-way Analysis of Variance; ANOVA) to examine differences in CORT between observer treatment (MG or Sham) and individual sex (Female or Male), and completed separate analyses for the chronic and acute observation treatments because measures of CORT can be affected by fine-scale differences in sampling time (Breuner et al. 1999), and we sampled each observation treatment several hours apart. We developed several possible models to test *a priori* to understand how CORT may change across treatments or sex: a null model of no changes in CORT; differences in CORT as a function of Treatment; differences in CORT as an additive function of Treatment + Sex; and differences in CORT as a function of the interaction between Treatment * Sex. Therefore, we adopted a model selection approach based on Akaike Information Criterion for small sample sizes (AICc) to make inference from these four competing hypotheses in each of the chronic and acute model sets (Burnham and Anderson 2004; Burnham et al. 2011). We also calculated evidence ratios using the “AICcmodavg” package (Mazerolle 2020) to compare closely supported models in our model sets when appropriate (Burnham and Anderson 2004). An evidence ratio provides the amount of times a given model is more parsimonious than a lower-ranked model. All analyses were performed using base R (version 4.2.1) within RStudio (R Core Team 2023). Plots were also prepared in R, using the “ggplot2” (Wickham 2016) and “ggpubr” packages (Kassambara 2023).

## Results

**Fig. 1:**
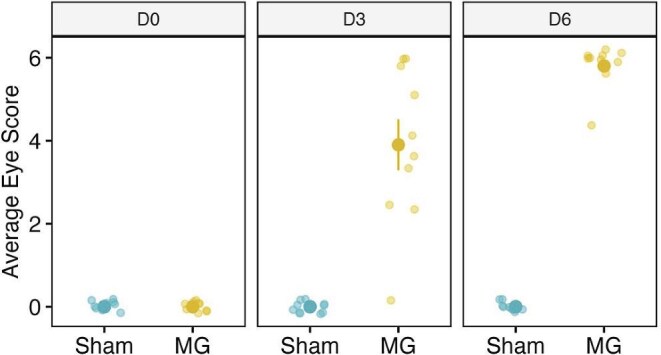
Plots of mean and standard error of eye inflammation score values for sham-infected or MG-infected stimulus canaries at day 0 (D0), day 3 (D3), and day 6 (D6) post-infection.

All 74 canaries (64 observers and 10 stimuli) had no evidence of displaying MG antibodies at arrival to our facilities and were assumed to be MG-naïve at the start of our experiments (Supplementary Material; MG antibody results). qPCR from observer eye swabs at experiment completion confirmed that observers remained MG-free throughout the experiment (Supplementary Material; MG qPCR results). While all MG-infected stimulus animals developed clinical signs of conjunctivital inflammation, with symptoms visible at day 3 for all but one animal, none of the sham-infected animals did (Fig. 1). The individual that did not show signs of infection on Day 3 did show signs of infection at Day 6.

### Chronic observation experiment

**Fig. 2:**
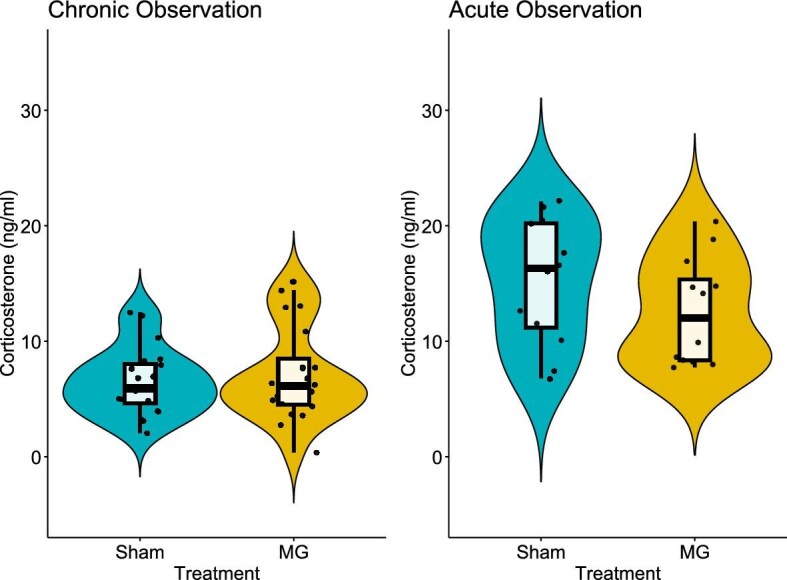
Boxplot and violin plot of corticosterone concentrations (CORT) from domestic canaries that observed MG-infected or sham-infected conspecifics for 6 days (chronic) or 1 h (acute). Boxplots show the median, the first and third quartiles (lower and upper hinges), and the smallest and largest values (lower and upper whiskers) no further than the interquartile range. Black circles represent individual bird corticosterone (ng/mL) values.

We found no support for an effect of stimulus infection treatment on observer corticosterone levels after 6 days of observation (Table 2, Fig. 2). The null model was overwhelmingly supported in our model set with a model weight (*w*) of 0.69. The second-best model did not improve model fit (−2log-likelihoods unchanged from the top model; Table 2) with the addition of one additional parameter (*k*) of treatment, indicating the presence of an uninformative “pretending” parameter, therefore that model is not considered for inference (Leroux 2019; Sutherland et al. 2023). The third best-supported model, an additive effect of sex and treatment, displayed some support with a ΔAICC of 4.71 (Table 2). However, the evidence ratio calculated between the Null model and the additive model indicated that the Null model was 10.54× more likely to be supported than the additive model.

**Table 2:** Model selection results showing univariate, additive, and interactive effects of sex and treatment on domestic canary corticosterone concentrations after chronically observing diseased or healthy conspecifics (treatment) over 6 days (Chronic Observation Experiment)

Model	*k*	AICc	Δ AICc	*w*	−2log(L)
Null	2	86.40	0.00	0.69	−41.04
Treatment	3	88.66	2.25	0.22	−40.99
Sex + Treatment	4	91.11	4.71	0.07	−40.98
Sex * Treatment	5	93.70	7.29	0.02	−40.97

### Acute observation experiment

The null model was the best supported model in our model set for the acute experiment (Table 3). CORT levels of observers between the sham and MG treatments of the acute experiment were similar (Fig. 2). The second-best model also contained a pretending variable that negligibly improved model fit (□−2log-likelihood <1). Like the results of the Chronic experiment, the third best model, the additive model of sex and treatment, had a low model weight of 0.10 (*w*) compared to the null model’s 0.53 (Table 3), and the evidence ratio between the two models indicate the null model is 5.6× more likely than the additive model.

**Table 3:** Model selection results showing univariate, additive, and interactive effects of sex and treatment on domestic canary corticosterone concentrations after acutely observing diseased or healthy conspecifics (treatment) for 1 h (Acute Observation Experiment)

Model	*k*	AICc	Δ AICc	*w*	−2log(L)
Null	2	150.10	0.00	0.53	−72.76
Treatment	3	150.93	0.83	0.35	−71.87
Sex + Treatment	4	153.55	3.45	0.10	−71.72
Sex*Treatment	5	156.68	6.58	0.02	−71.67

## Discussion

We tested if healthy canaries would exhibit altered CORT responses when subjected to observing diseased conspecifics. Our study did not find evidence that CORT increased in the presence of diseased conspecifics. This is in contrast with previous studies in laboratory mice and rats (Curno et al. 2009; Hamasato et al. 2014; Hamasato et al. 2017; Castany et al. 2024), where CORT increased when healthy rodents were exposed to parasitized, cancerous, or lipopolysaccharide-treated conspecifics. We propose three possible explanations for a lack of an effect of perceived disease risk on CORT: (1) a different time-course of CORT increase; (2) experimental limitations or choices; and (3) a true lack of participation of CORT in modulating changes in immune responses during high infection risk situations.

In our chronic experiment, we chose to measure CORT on D6 because Love et al. (2021) documented substantial differences at this time point in immune responses (e.g., increased heterophil: lymphocyte ratios; H:L ratios) between canaries observing MG-infected and sham-infected conspecifics in a similar study. Increases in heterophils/neutrophils are known to occur with increases in CORT (Davis and Maney 2018) and may partly be responsible for this response. However, we did not observe differences in CORT between observers of MG or sham-infected conspecifics at D6. If CORT is involved in increasing H:L ratios as observed in this host–pathogen system (Love et al. 2021), then it is possible that any elevation in CORT to trigger these immune responses occurred early on, before quickly dissipating by D6. In fact, Love et al. (2021) observed minor differences in H:L ratios as early as D3 in their study, signaling the beginning of immune activity. Therefore, it is possible that we missed a small window when CORT was elevated to promote this immune response.

Results from our acute experiment were similar, showing no evidence for an effect of treatment or sex on observer CORT. Birds across both treatments in the acute experiment appeared to potentially display higher CORT responses than those in the chronic experiment, though we did not specifically test for statistical differences between experiments due to large discrepancies in sample sizes. It is possible that the reduced time for acclimation of birds to the experimental room (only one overnight), and their change from communal to single housing, elicited an increased CORT response in all observers that prevented us from detecting a possible response to the MG-infected stimulus birds. For example, mice that changed housing rooms displayed higher CORT 24 h later, as did those that were shipped and placed in new housing (Olfe et al. 2010). Future studies should use an experimental design that allows the observers to acclimate to the testing room for longer periods of time, and then introduce the stimulus birds at peak conjunctival symptoms to the experimental room instead. In addition, the smaller sample sizes in our acute experiment (12 birds) may have prohibited us from detecting differences between treatments, should they occur. It is also possible that it takes longer than 1 h of observation to trigger a CORT response, so additional time points between 1 h and D3, for example, could be tested in the future.

Differences in experimental design from previous studies may have also reduced the potential to measure the effects of infection risk. We introduced a biosecurity measure across both experiments to limit the possibility of aerosol transmission from MG-infected birds to uninfected observers that prior studies omitted. Our use of a HEPA filter with secondary carbon filter may have reduced any olfactory cues of infection in the experimental rooms. While pathogen avoidance via olfactory cues is well documented in mammals (e.g., Sharp et al. 2015 and Kavaliers et al. 2022), to our knowledge, it is entirely unstudied in birds and no evidence exists for this phenomenon. Therefore, we do not believe the HEPA filter interfered with birds’ perception of infection risk in our experiment. Furthermore, in an identical experimental design using a different batch of canaries, we documented changes in activity time of individuals observing MG-infected vs. sham-infected birds (Supplemental Information). These behavioral changes by observer birds provide evidence that the experimental design we employed in the present study did not obstruct observers’ detection of heightened infection risk.

Lastly, it is possible that healthy canaries observing sick conspecifics do not increase their CORT and that the mechanisms that lead to changes in immunity following observation are not directly or solely connected to CORT levels. For example, a study in female Japanese quail (*Coturnix japonica*) exposed to lipopolysaccharide-treated males for a few hours suggested possible changes in molecular pathways downstream of CORT (Gormally and Lopes 2023). While Love et al. (2021) documented increased H:L ratios which are known to increase with elevated CORT (Davis and Maney 2018), other predictions for the effects of CORT on immune responses were not supported by their results. For example, elevated CORT is predicted to suppress complement activity (Gao and Deviche 2019) and increase IL1β and IL6 (Takaki et al. 1994, Dhabhar 2009), but research in canaries has shown increased complement activity and a decrease in IL1β and IL6 (Love et al. 2021), responses opposite of what is predicted. Therefore, we suggest that other hormonal mechanisms may be involved in immune priming other than CORT alone. For example, prolactin is also produced during the HPA stress response from the pituitary (Nelson and Kriegsfeld 2017). Prolactin exhibits immunomodulating effects in several bird species correlated with increased phagocytosis (Rodríguez et al. 1996) and bacterial killing ability (Rubenstein et al. 2008). Norepinephrine is released much quicker than CORT via the sympathetic nervous system during acute stress and is known to increase certain circulating immune cells (Dhabhar et al. 2012) and increase TNF-α (Huang et al. 2012), a cytokine that influences the strength of immune responses (Murphy et al. 2022). However other studies document the inhibitory effects of norepinephrine on immune responses under certain conditions (Cao and Lawrence 2002, Yano et al. 2010). Thus, the relationships between various hormones involved in HPA activation and immune function are ultimately complex (Sapolsky 1992; Adamo 2008; Matson et al. 2012), and warrant further focus in the context of the effects of infection risk on immune responses.

In conclusion, our study found no infection risk induced elevation in CORT either at 1 h or after 6 days of having visual access to diseased conspecifics. We believe it is still possible for CORT to be modulated by disease risk at other time points, and that, in addition to this time course aspect, other components of the stress response (such as prolactin and norepinephrine) would be worth quantifying in future studies. Considering the potential link between CORT and eye-pathology (Adelman et al. 2015), and that increased eye-pathology severity correlates with MG fomite deposition (Adelman et al. 2013), there remains significant need for understanding the effects of CORT on host–pathogen dynamics and disease transmission in the avian-MG system. Given the relationships between the stress response and the immune response, continuing to study whether infection risk elicits a stress response is critical for understanding how hosts perceive their risk of infection.

## Supplementary Material

obag008_Supplemental_File

## Data Availability

All data used for analysis in this manuscript are provided within the Supplementary Material.
